# N-α-PGP and PGP, potential biomarkers and therapeutic targets for COPD

**DOI:** 10.1186/1465-9921-10-38

**Published:** 2009-05-18

**Authors:** Philip O'Reilly, Patricia L Jackson, Brett Noerager, Suzanne Parker, Mark Dransfield, Amit Gaggar, J Edwin Blalock

**Affiliations:** 1 Division of Pulmonary and Critical Care Medicine, University of Alabama at Birmingham, THT 422, 1900 University Blvd, Birmingham, Alabama 35294-0006, USA; 2 Department of Pathology, University of Alabama at Birmingham, MCLM 894, 1918 University Blvd, Birmingham, Alabama 35294-0005, USA; 3 Department of Physiology and Biophysics, University of Alabama at Birmingham, MCLM 894, 1918 University Blvd, Birmingham, Alabama 35294-0005, USA; 4 Division of Pharmacology and Pathophysiology, Department of Pharmaceutical Sciences, Faculty of Science, Utrecht University, Utrecht, the Netherlands

## Abstract

**Background:**

Chronic obstructive pulmonary disease (COPD) is a common respiratory disorder for which new diagnostic and therapeutic approaches are required. Hallmarks of COPD are matrix destruction and neutrophilic airway inflammation in the lung. We have previously described two tri-peptides, N-α-PGP and PGP, which are collagen fragments and neutrophil chemoattractants. In this study, we investigate if N-α-PGP and PGP are biomarkers and potential therapeutic targets for COPD.

**Methods:**

Induced sputum samples from COPD patients, healthy controls and asthmatics were examined for levels of N-α-PGP and PGP using mass spectrometry and for the ability to generate PGP *de novo *from collagen. Proteases important in PGP generation in the lung were identified by the use of specific inhibitors in the PGP generation assay and by instillation of proteases into mouse lungs. Serum levels of PGP were compared between COPD patients and controls.

**Results:**

N-α-PGP was detected in most COPD sputum samples but in no asthmatics or controls. PGP was detected in a few controls and in all COPD sputum samples, where it correlated with levels of myeloperoxidase. COPD sputum samples had the ability to generate N-α-PGP and PGP *de novo *from collagen. PGP generation by COPD sputum was blocked by inhibitors of matrix metalloproteases (MMP's) 1 and 9 and prolyl endopeptidase. MMP's 1 and 9 and prolyl endopeptidase acted synergistically to generate PGP *in vivo *when instilled into mouse lungs. Serum levels of PGP were also significantly higher in COPD patients than in controls

**Conclusion:**

N-α-PGP and PGP may represent novel diagnostic tests and biomarkers for COPD. Inhibition of this pathway may provide novel therapies for COPD directed at the chronic, neutrophilic, airway inflammation which underlies disease progression.

## Background

Chronic obstructive pulmonary disease (COPD) is a significant and growing healthcare problem in the United States and worldwide [1,2]. Currently, there are no therapies for COPD that substantially alter its natural history or improve outcomes [3]. A major impediment to COPD research and management is the lack of readily measurable biomarkers that correlate with disease severity and outcome [4,5].

Chronic, neutrophilic airway inflammation is central to disease pathology and progression in COPD [6] but the mechanisms that underlie this inflammation are incompletely understood. Inhibiting classic pathways, such as interleukin-8 and leukotriene B4, blocks less than half of the neutrophil chemotactic activity of COPD sputum [7], indicating that other, as yet unidentified, chemoattractants are likely involved. Improved understanding of the neutrophilic airway inflammation of COPD would provide novel biomarkers and therapies directed, for the first time, at the underlying mechanism of disease.

A hallmark of COPD is emphysema, defined as dilation and destruction of lung parenchyma distal to the terminal bronchiole [8]. One theory of emphysema causation is over-activation of proteases secreted by inflammatory cells which degrade extracellular matrix components and destroy the alveolar epithelium [9]. Proteases implicated in COPD include human neutrophil elastase (HNE) and the matrix metalloproteases (MMP's), a family of zinc-dependent metalloendopeptidases [10]. Mice deficient in HNE or MMP-12 demonstrate decreased airspace enlargement and inflammatory cell infiltration after long-term exposure to cigarette smoke [11,12], COPD patients demonstrate increased activity of HNE and MMP's, including MMP-1 and 9, in their lungs [13-15], and mice which over-express MMP-1 develop adult onset emphysema [16]. Importantly, it has been recognized for more that 20 years that fragments of matrix proteins, generated by protease activity, have chemotactic activity for neutrophils and monocytes and may also be pro-inflammatory [17-19]. The role of these fragments in lung inflammation in *vivo *has recently become evident. For example, inhibiting the monocyte chemotactic activity of elastin fragments reduces experimental emphysema in mice [20].

We have recently described a potentially new pathway that signals neutrophil infiltration followed by damage to the airways and may represent a novel etiology as well as diagnostic and therapeutic target for chronic airway diseases [21]. In 1995, Pfister and colleagues demonstrated that alkali degradation of whole cornea generated a tri-peptide, N-acetyl-proline-glycine-proline (N-α-PGP) that is chemotactic for neutrophils and likely results from hydrolysis of collagen [22]. Injection of N-α-PGP into normal corneas recapitulated the neutrophilic inflammation seen in alkali injury to the eye [23]. Instillation of N-α-PGP into the lungs of mice caused a marked recruitment of neutrophils to the airways and chronic airway exposure caused COPD-like pathology with alveolar enlargement and right ventricular hypertrophy [21]. The neutrophil chemotactic activity of N-α-PGP is exerted through binding of CXC receptors and is due to a marked structural homology to ELR-positive CXC chemokines [21]. Generation of PGP is due to the action of MMP's and prolyl endopeptidase (PE) on collagen in a step-wise fashion [24]. N-α-PGP and PGP, which is also a neutrophil chemoattractant, are biomarkers for cystic fibrosis (CF) and increase further during exacerbations [24]

In this study, we demonstrate that N-α-PGP and PGP are biomarkers for COPD and are generated by an enzymatic cascade involving MMP's and PE. PGP generation by COPD sputum is blocked by inhibitors of MMP's and PE, which could provide the basis for novel therapies directed at COPD neutrophilic, airway inflammation.

## Methods

### Patient Populations and Sputum Collection

COPD patients were recruited from the UAB Lung Health Center database of COPD patients and had irreversible airflow obstruction (FEV_1_/FVC < 70%). COPD patients had FEV_1 _values ranging from 27% to 83% predicted with a median of 47% predicted. The majority had severe disease (FEV_1 _< 50% predicted) according to the GOLD criteria [3]. COPD patients were clinically stable and had not experienced an exacerbation of their disease for at least three months prior to recruitment. Asthmatic sputum samples were kindly provided by Dr A. Hastie (Wake Forest University, North Carolina, USA) and were obtained from subjects participating in the Severe Asthma Research Study (National Institutes of Health, Bethesda, Maryland, USA). Patients with severe asthma met the criteria for the consensus definition for refractory asthma of the American Thoracic Society, which requires signs of ongoing poor asthma control (daily symptoms, additional medication use, high health care utilization, abnormal lung function) despite treatment with high doses of corticosteroids [25]. Normal controls were non-smokers with no history of lung disease. Approval was obtained from the Institutional Review board at UAB prior to conducting these studies. All subjects provided informed consent. Samples and health information were labeled using unique identifiers to protect subject confidentiality. Sputum was obtained by induction using 3% saline according to standard methodology [26]. Sputum samples were collected on ice, diluted 1:1 with 0.9% saline and stored at -80°C for later analysis.

### Electrospray ionization-liquid chromatrography-mass spec/mass spec (ESI-LC-MS/MS)

Sputum and serum samples were prepared for analysis by mass spectrometry as previously [24,27]. PGP and N-α-PGP were measured simultaneously using a MDS Sciex API-4000 spectrometer (Aplied Biosystems) equipped with a Shimadzu HPLC. HPLC was performed using a 2.1 × 150 mm Develosi C30 column (buffer A: 0.1% formic acid, buffer B: acetonitrile plus 0.1% formic acid; 80% buffer A/20% buffer B from 0 to 0.6 min, 0% buffer A/100% buffer B from 0.6 to 5 min). Background was removed by flushing with 100% isopropanol/0.1% formic acid. Positive electrospray mass transitions were at 270-70 and 270-116 for PGP and 312-140 and 312-112 for N-α-PGP. The R^2 ^value for the calibration curves for PGP was 0.988 and the detection limit was 10 pg/ml. The intra- and inter-assay variabilities were 7.8% and 12.6%, respectively.

### Myeloperoxidase (MPO) assay

This was performed using a commercially available activity assay (Calbiochem, San Diego, California, USA). Samples and standards were added to wells coated with a polyclonal antibody to human MPO and incubated for two hours. Detection reagent (tetramethylbenzidine and hydrogen peroxidase) was added for one hour and absorbance read at 450 nm. Activity was converted to ng/ml active MPO using a standard curve.

### Ex Vivo Collagen Assay

One hundred microliters of saline-diluted sputum was incubated with extensively dialyzed, intact type I collagen (50 μl, 1 mg/ml, Sigma Aldrich) for 24 h at 37°C and 5% CO_2_. After incubation, samples were filtered through a 10-kDa filter, washed with 20 μl of 1 N HCl, and analyzed using ESI- LC-MS/MS for levels of PGP and N-α-PGP. Amounts of PGP and N-α-PGP generated by each sputum sample from collagen were determined by comparison with sputum incubated with PBS. For inhibitor studies, the six most active sputum samples were incubated with selected protease inhibitors or with azithromycin, a macrolide antibiotic, before collagen was added. Inhibitors are listed in Table 1.

**Table 1:** Protease Inhibitors used in *ex vivo *collagen assay

Enzyme Inhibited	Chemical Composition*(Source)*	Efficacy	Ref.
PE	Z-prolyl prolinal (*Calbiochem*)	K_i _= 500 pM	[36]
MMP-9	C_27_H_33_N_3_O_5_S (*Calbiochem*)	IC_50 _= 5 nM	[37]
MMP-1	(4-(4-(Methanesulfonamido)phenoxy)phenylsulfonyl) methyloxirane (*Calbiochem*)	K_i _= 45 μM	[38]
HNE	N-(2-(4-(2,2-dimethylpropionyloxy)phenylsulfonylamino)benzoyl) aminoacetic acid N-(*o*-(*p*-pivaloyloxybenzene)sulfonylamino benzoyl)glycine (*Calbiochem*)	IC_50 _= 50 nM	[39]

### MMP-1 and MMP-9 assays

These were performed using commercially available activity assays (R and D systems, Minneapolis, Minnesota, USA). Samples and standards were added to wells coated with a monoclonal antibody to MMP-1 or MMP-9 and incubated for two hours at room temperature. A fluorogenic substrate (Fluor-Pro-Leu-Gly-Leu-Ala-Arg-NH_2_) was added and the plate incubated for 18 hrs at 37°C. Activity was quantified using a spectrophotometer with excitation and emission wavelengths of 320 and 450 nm respectively and converted to ng/ml active MMP using a standard curve.

### Administration of proteases to mice

The animal protocol for protease administration was approved by the University of Alabama, Birmingham Institutional Animal Care and Use Committee. MMP-1 (55.6 μg/kg) and/or MMP-9 (55.6 μg/kg) with or without PE (18.4 mg/kg) were administered intra-tracheally to 4–6 week old Balb/c mice in a total volume of 50 μl. MMP's were preactivated with 1 mM aminophenylmercuric acid for 2 hrs at 37°C before administration. After 24 hrs, mice were euthanized with phenobarbital and broncho-alveolar lavage was performed with four 1 ml aliquots of cold PBS. PGP was quantified in BAL fluid by ESI-LC-MS/MS.

### Statistical Analysis

Descriptive statistics (mean and standard error of the mean) were computed for all study variables of interest. Spearman's rank correlation coefficient (r) was used to test for relationships between variables in sputum samples. The two group t test was used for comparisons between groups. Statistical analyses were performed using SAS software (version 9.3; SAS Institute Inc., Cary, North Carolina, USA). P values < 0.05 were considered statistically significant.

## Results

### N-α-PGP and PGP are detected in sputum from COPD patients

**Figure 1:**
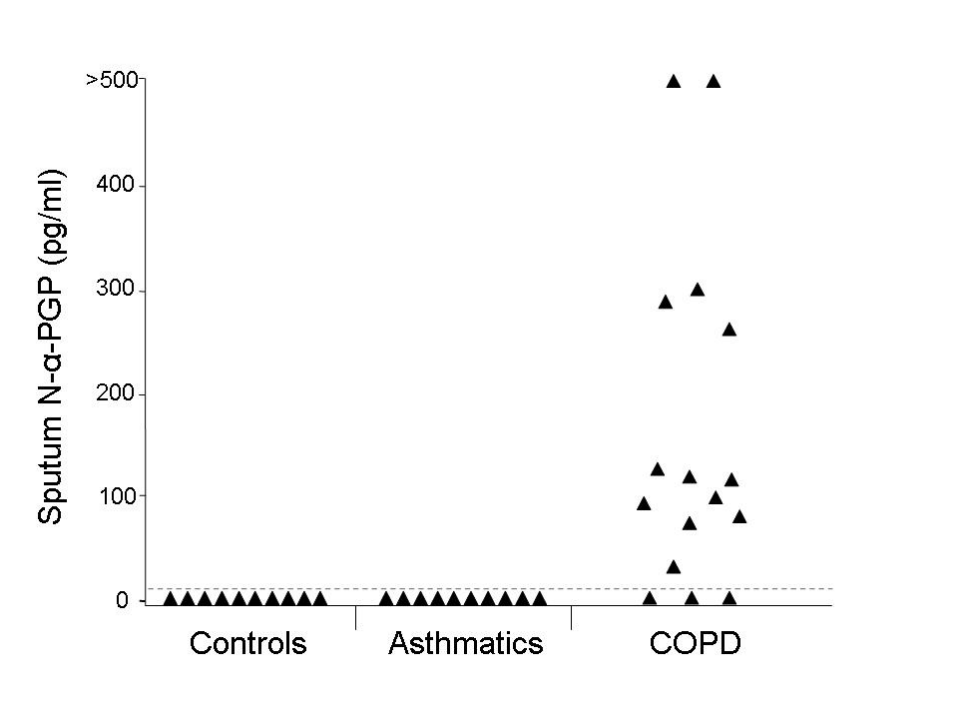
**N-α-PGP may be a sputum biomarker for COPD**. Sputum from 13 of 16 COPD patients but from no asthmatics (0/10) or controls (0/10) contains N-α-PGP. N-α-PGP was measured by ESI-LC-MS/MS with a limit of detection of 10 pg/ml (dashed line).

**Figure 2:**
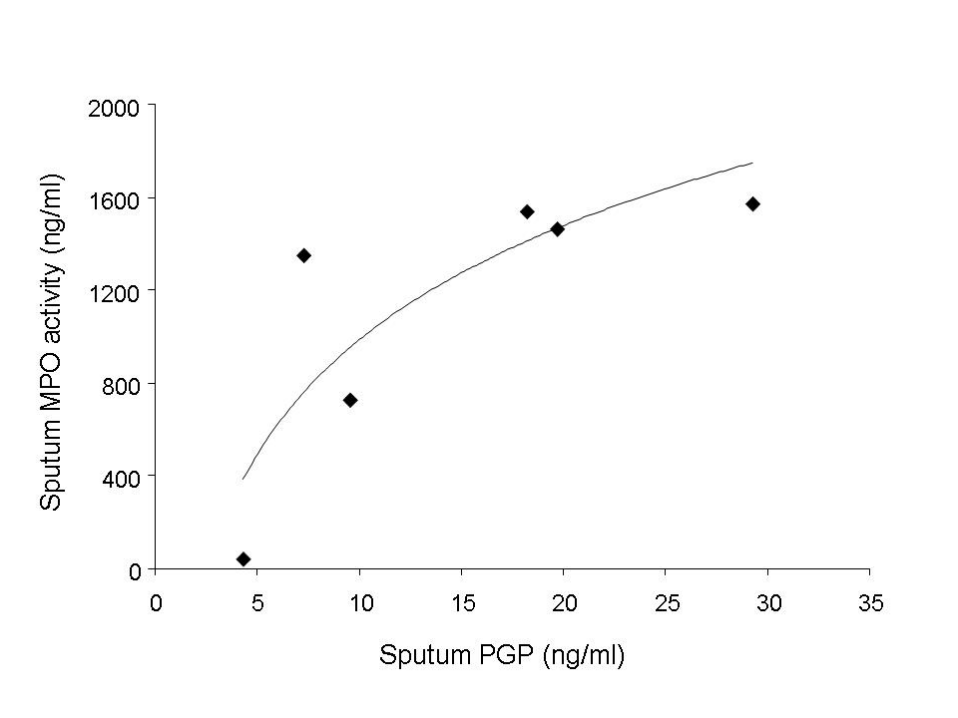
**PGP correlates with MPO activity in COPD sputum**. MPO activity in sputum from six COPD patients was measured using an activity ELISA and correlated with PGP. The Spearman rank correlation coefficient of PGP with MPO was 0.89 (p = 0.02). The neutrophil chemoattractant activity of PGP approaches a maximum at 30 ng/ml of PGP.

Induced sputum from ten controls, ten asthmatics and 16 COPD patients was examined for N-α-PGP by ESI-LC-MS/MS. Sputum from 13 of 16 COPD patients (81%) was positive for N-α-PGP above our limit of detection of 10 pg/ml but all asthmatic and control sputum samples were negative (Fig. 1). N-α-PGP levels in COPD sputum were 163 ± 41 pg/ml. Non-acetylated PGP was detected in all COPD sputum samples (16/16) and in a minority of controls (3/10) but levels were higher in the COPD patients (58 ± 12 ng/ml vs. 22 ± 12 ng/ml, p < 0.05). Consequently, N-α-PGP and PGP may be biomarkers that distinguish COPD patients from asthmatics and healthy controls. There was no correlation between lung function (% predicted FEV_1_) and N-α-PGP or PGP levels in the COPD patients. PGP levels in COPD sputum correlated with MPO activity, an index of neutrophilic inflammation, at lower levels of PGP (Fig. 2). This supports a role for PGP as a neutrophil chemoattractant in COPD. Of note, asthmatic samples were obtained from severe asthmatics and contained significant amounts of neutrophils (0.07 – 1.8 × 10^6^/ml), which must be recruited to the airways by chemotactic factors other than N-α-PGP.

### COPD sputum generates PGP de novo from collagen

**Figure 3:**
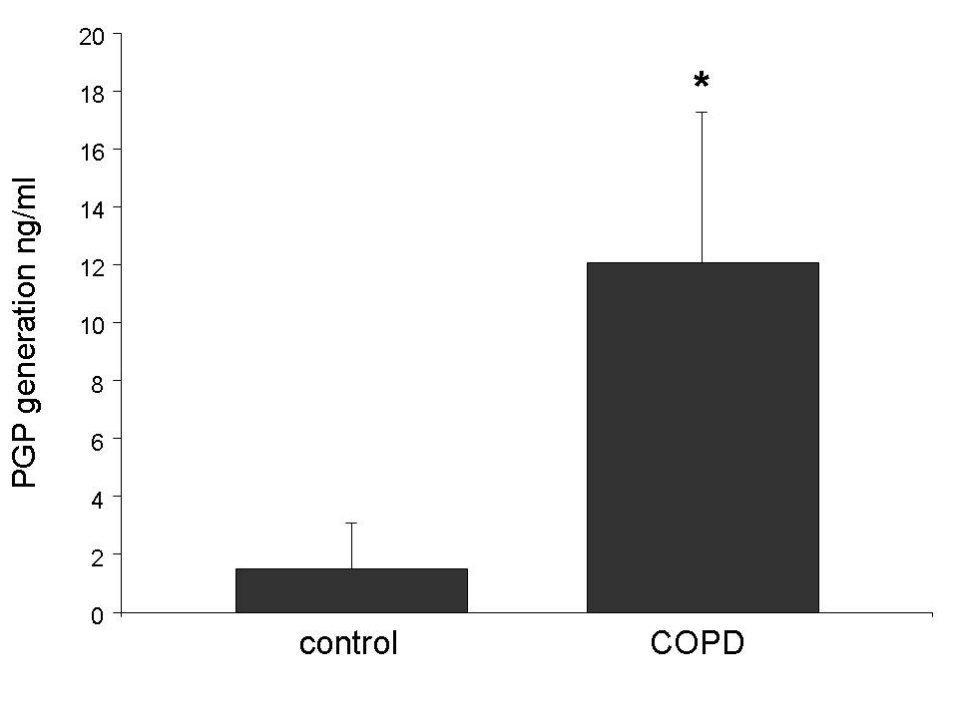
**COPD sputum generates PGP *de novo *from collagen**. Sputum from eight COPD patients and ten controls was incubated with Type I collagen for 24 hrs at 37°C. PGP in samples was measured using ESI-LC-MS/MS and expressed as amount of PGP generated (ng/ml) compared to sputum incubated with PBS. COPD sputum samples generated significantly greater amounts of PGP than control samples (* p < 0.05). Results are presented as mean ± SEM. Comparison between COPD and control sputum was performed using the two group t test.

We examined the ability of COPD sputum to generate PGP from collagen *ex vivo*. Induced sputum from eight COPD patients and ten controls was incubated overnight with type I collagen which contained no PGP. The amount of PGP generated by each sputum sample was determined by comparison with sputum incubated with PBS. Type I collagen was used as it is the predominant collagen found in the airways [28]. Figure 3 shows that sputum from COPD patients generated much greater amounts of PGP from collagen than sputum from controls, which generated small amounts of PGP. This indicates that COPD sputum contains the enzymatic activity necessary to generate PGP *de novo *from intact collagen. Much smaller amounts of N-α-PGP were generated (data not shown), indicating that acetylation is the likely rate-limiting reaction for N-α-PGP formation.

### MMP and PE activity underlies PGP generation by COPD sputum

We sought to identify the proteases responsible for PGP generation from collagen by COPD sputum. We focused on MMP-1, MMP-9 and HNE, which have been detected in elevated amounts in COPD sputum and can degrade matrix proteins, and on PE, which to our knowledge is the only enzyme that can generate PGP from collagen [24]. As PE is an oligopeptidase, it must act on substrates previously generated from collagen by the action of other proteases.

**Figure 4:**
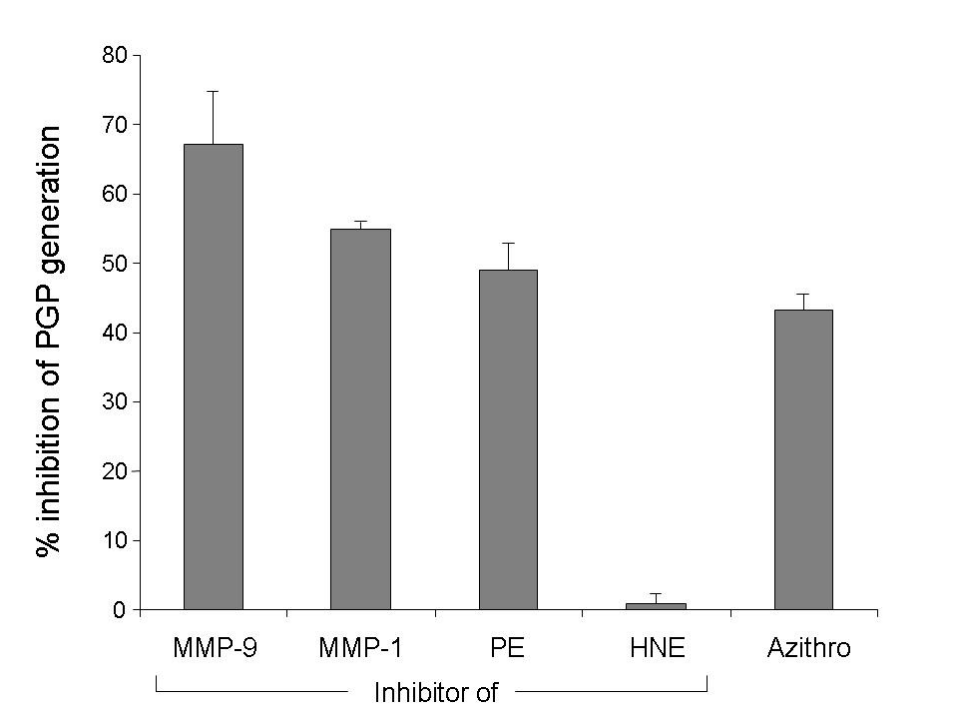
**MMP's and PE but not HNE underlie PGP generation by COPD sputum**. Inhibitors of MMP-1, MMP-9, PE and HNE and azithromycin, a macrolide antibiotic, were included in the assay of PGP generation by COPD sputum. MMP inhibitors were used at 50 μM, PE inhibitor and azithromycin at 100 μM and HNE inhibitor at 1 μM. MMP-1, MMP-9 and PE inhibitors and azithromycin reduced PGP generation by COPD sputum whereas inhibition of HNE had no effect. Experiments were performed twice using pooled sputum from six COPD patients. Results are shown as percent inhibition of PGP production compared to sputum without inhibitors and are presented as mean ± SEM (n = 2).

We included inhibitors of MMP-1, MMP-9, HNE and PE in our *ex vivo *PGP generation assay. We also included azithromycin, a macrolide antibiotic, because of its known, anti-inflammatory properties in chronic, respiratory diseases [29]. The MMP-9 inhibitor was most effective at reducing PGP generation (Fig. 4). Inhibition of MMP-1 and PE also reduced PGP generation but inhibition of HNE had no effect. The lower efficacy of the MMP-1 inhibitor at inhibiting PGP production may be explained by its lower affinity (Table 1). These data support a role for MMP's and PE in PGP generation in COPD. Azithromycin also reduced PGP generation from collagen by COPD sputum.

### MMP-1 and MMP-9 act synergistically to increase PGP production in the lung

**Figure 5:**
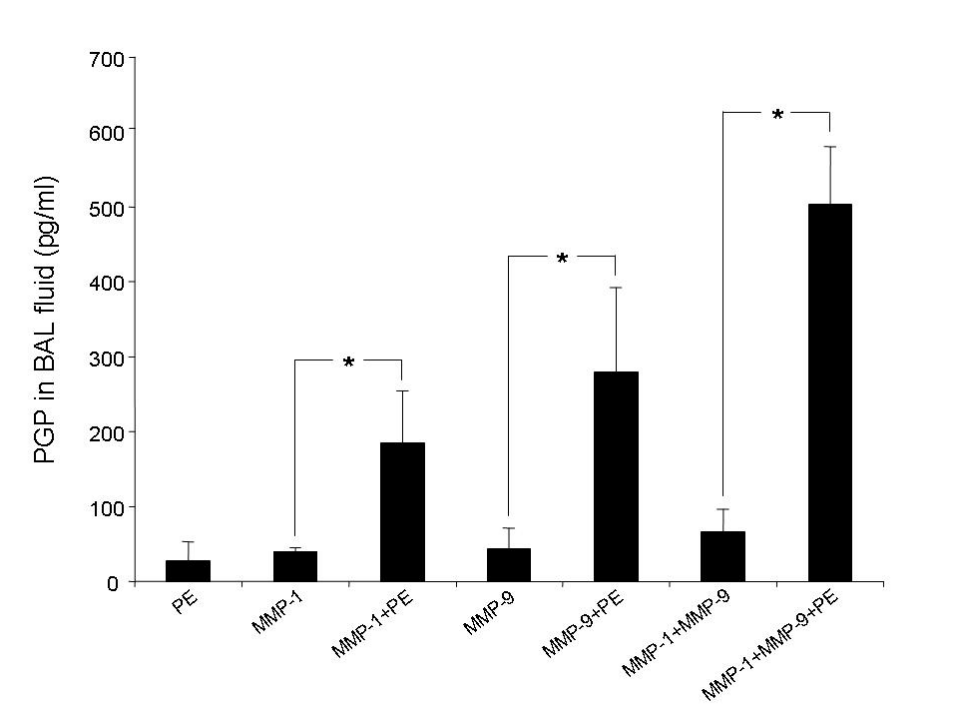
**MMP-1, MMP-9 and PE act synergistically to generate PGP *in vivo***. Protease combinations were administered intratracheally to mice (4 to 7 per group) and BAL fluid collected 24 hours later. PGP levels in BAL fluid were measured by ESI-LC-MS/MS. Addition of PE to MMP's greatly increased PGP production over MMP's alone (* p < 0.05 vs. MMP-1, MMP-9 or MMP-1 + MMP-9). MMP-1 and MMP-9 combined with PE generated more PGP than either MMP-1 or MMP-9 alone with PE (p < 0.05). Results are presented as mean ± SEM. Comparisons between groups were performed using the two group t test.

We measured MMP-1 and MMP-9 activity in sputum from eight COPD patients and correlated them with levels of PGP. MMP-1 and MMP-9 were detected in all samples (Table 2). MMP-9 activity was much higher than MMP-1 activity (186 ± 107 ng/ml vs. 6 ± 0.6 ng/ml). PGP levels did not correlate significantly with MMP-9 (r = 0.14, p = 0.74) or MMP-1 (r = 0.62, p = 0.10) activity. However, when we adjusted for MMP-9, the correlation coefficient of PGP with MMP-1 activity approached statistical significance (r = 0.72, p = 0.065 by partial Spearman correlation using n – 3 degrees of freedom). This suggests that total rather than individual MMP activity is important in PGP generation in COPD. To test the idea that MMP-1 and MMP-9 combine to increase PGP generation in the lung, we administered MMP-1 and/or MMP-9 to mice intratracheally with and without PE and measured PGP 24 hours later in BAL fluid. MMP-1, MMP-9 and PE alone generated small amounts of PGP. PGP production was greatly increased by the addition of PE to either MMP-1 or MMP-9. Combining MMP-1 and MMP-9 together with PE significantly increased PGP generation over either MMP-1 or MMP-9 with PE (Fig. 5). These data support the idea that total MMP-1 and MMP-9 activity together with PE is important in generation of PGP from collagen in the lung and concur with our results in COPD sputum.

**Table 2:** PGP and MMP levels per subject

Subject #	PGP ng/ml	MMP-1 ng/ml	MMP-9 ng/ml
1	4.28	5.75	12
2	7.2	4.74	85.8
3	9.48	4.41	61.4
4	18.16	4.51	286.4
5	19.64	5.16	82.2
6	29.2	8.83	36
7	130	5.88	903.6
8	145.2	8.72	22

### PGP is detected in serum of COPD patients

**Figure 6:**
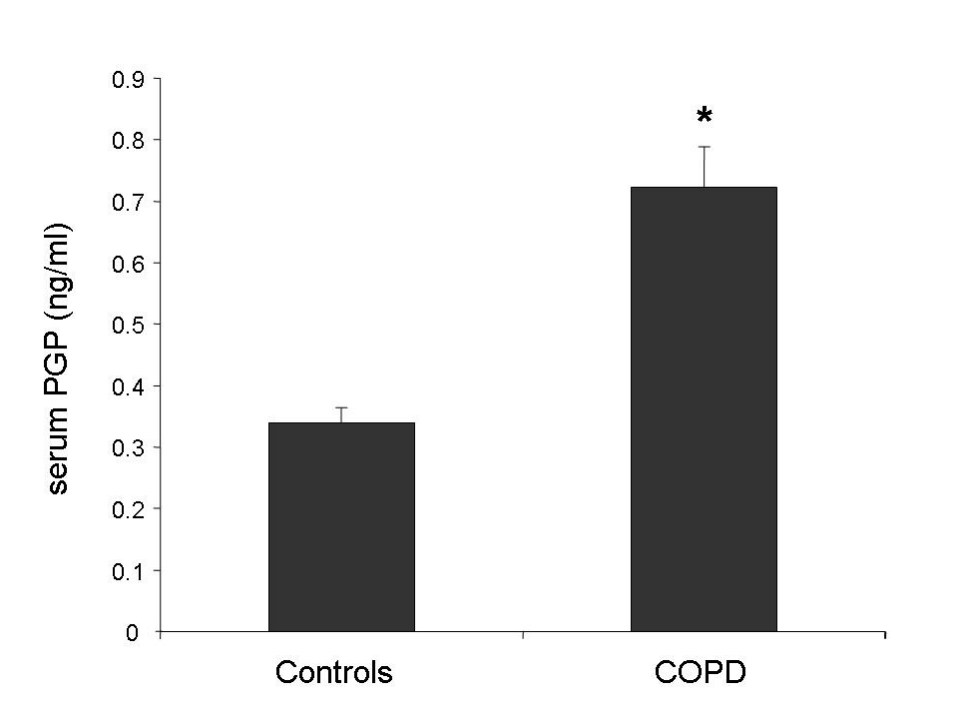
**PGP may be a serum biomarker for COPD**. PGP levels in serum were measured in serum of eight COPD patients and five controls by ESI-LC-MS/MS. Levels were higher in the COPD patients (* p < 0.01). Results are presented as mean ± SEM. Comparison between COPD and control serum was performed using the two group t test.

We explored the potential of PGP to be a serum biomarker for COPD which would greatly increase its clinical usefulness. To this end, we adapted our ESI-LC-MS/MS protocol to quantify PGP in serum of COPD patients and healthy controls [27]. We found that PGP levels were more than twice as high in serum of COPD patients as in controls (Fig. 6). We measured levels of PGP in sputum and serum obtained simultaneously from six COPD patients. The correlation coefficient (Spearman) of sputum with serum PGP in this small group was 0.71 (p = 0.11). Altogether, these data suggest the potential for PGP to be a serum biomarker for COPD that reflects inflammation in the lung.

## Discussion

N-α-PGP and non-acetylated PGP are breakdown products of collagen generated by proteases and are neutrophil chemoattractants. Here we report that N-α-PGP and PGP may be biomarkers for COPD which is characterized by chronic, neutrophilic, airway inflammation and extensive matrix remodeling. N-α-PGP was detected in most COPD patients but in no asthmatics or controls whereas PGP was detected in all COPD patients and a minority of controls. This suggests that PGP may be present in the healthy lung as a result of normal collagen turnover whereas N-α-PGP is always a biomarker of disease. Acetylation of PGP might represent an important step in the pathogenesis of COPD and other chronic neutrophilic lung diseases. This is not surprising as N-α-PGP is the more potent neutrophil chemoattractant [30]. However, PGP is present in much greater amounts in sputum (ng vs. pg) and may be responsible for as much neutrophil chemotactic activity *in vivo*. The correlation between PGP and MPO in COPD sputum (Fig. 2) could mean that PGP is acting as a neutrophil chemoattractant or, alternatively, that it is generated by neutrophilic inflammation. This is possible as neutrophils contain many of the proteases involved in generation of PGP, including MMP's [31], and neutrophils were required for PGP production in a mouse model of LPS-induced keratitis [32]. In this way, N-α-PGP and PGP could potentially feed back to stimulate their own production.

Levels of N-α-PGP and PGP detected in this study were much lower than we previously detected in CF sputum [24]. However, our subjects were stable outpatients whereas the CF patients were experiencing exacerbations. It is likely that N-α-PGP and PGP levels would increase further during exacerbations of COPD. Also, our COPD patients had advanced disease and most PGP generation might occur early when matrix destruction is most active. This might explain our inability to correlate levels of N-α-PGP/PGP with pulmonary function in our COPD group. COPD is a heterogeneous disease with degrees of airway inflammation and emphysema differing greatly between individual patients. Definitive answers to these questions will await a larger study of COPD patients at different stages of disease and with different phenotypes.

Sputum from COPD patients has the ability to generate PGP *de novo *from collagen. Through the use of specific inhibitors, we demonstrate a role for MMP's-1 and 9 and PE in this process. As PE is an oligopeptidase, we believe it likely acts on substrates generated from MMP digestion of intact collagen to generate PGP. Our data suggest that MMP's-1 and 9 act together to generate this substrate. Consistent with this idea, MMP-1 and MMP-9 had an additive effect on PGP generation in mouse lungs (Fig. 5). Although MMP-12 and NE are prominently associated with COPD in cigarette smoke-exposed mice, they are not involved in the generation of PGP. We have recently found that PGP causes neutrophils to degranulate and release activated MMP-9 and NE (our data not shown). NE, in turn, has been shown to be required for neutrophil and macrophage recruitment and expression of active MMP-12 in the lungs of cigarette smoke-exposed mice [12]. In this way, PGP may be an upstream regulator of MMP-12 and NE expression, which contribute to monocyte and neutrophil recruitment and damage to cigarette smoke-exposed lungs.

COPD is greatly under-diagnosed and most patients are detected at an advanced stage when irreversible lung destruction has already occurred. As N-α-PGP and PGP are generated by matrix destruction in the lung, they might identify those smokers at risk of developing COPD many years before clinical, spirometric or radiographic signs appear. This would permit therapeutic and preventive measures to be instituted much earlier. In support of this idea, we have found that a subgroup of healthy smokers contain N-α-PGP in their exhaled breath condensate [33]. A biomarker that could distinguish COPD patients from severe asthmatics, many of whom have irreversible airflow obstruction and neutrophilic airway inflammation, would be of benefit. Although PGP did not correlate with FEV_1_, it correlated with MPO, an index of neutrophilic airway inflammation, which correlates with disease severity and rate of decline in pulmonary function in COPD. Such a biomarker would be of benefit as an endpoint in clinical trials, permitting them to be conducted faster and with fewer subjects than endpoints such as exacerbation frequency or FEV_1 _decline. PGP is detected in serum, where it is elevated in COPD patients compared to healthy controls. If serum PGP correlates with inflammation in the lung, this would greatly increase its usefulness as a biomarker

As N-α-PGP and PGP are neutrophil chemoattractants, reducing neutrophil recruitment to the airways by antagonizing them becomes an attractive therapeutic approach. In support of this idea, PGP antagonists prevented the induction of pulmonary emphysema and right ventricular hypertrophy in mice caused by chronic administration of LPS [34]. The therapeutic effect of azithromycin in chronic, neutrophilic lung diseases may in part be explained by effects on PGP generation (Fig. 4). We suspect that this is due to inhibition of MMP-9 and perhaps other MMP's, which is a known property of macrolide antibiotics [35]. Additional MMP and PE inhibitors have potential to be therapeutics for COPD given their role in PGP generation.

## Conclusion

These findings support the idea that N-α-PGP and PGP are novel biomarkers and therapeutic targets for COPD. Necessary future directions include measuring N-α-PGP and PGP in a larger cohort of COPD patients and controls, correlating them with clinical parameters such as smoking history, severity of airflow obstruction and degree of emphysema and establishing the reproducibility of N-α-PGP and PGP measurements over time. Inhibition of N-α-PGP and PGP or their generating enzymes could provide the basis for novel therapeutics directed at the neutrophilic, airway inflammation that underlies COPD pathogenesis and progression.

## Competing interests

The authors declare that they have no competing interests.

## Authors' contributions

PO'R processed the sputum samples, carried out data analysis and drafted the manuscript; PLJ performed the mass spectrometric analyses and collagen digestion assays; BN performed the animal studies; SP and MD provided the sputum and serum samples and provided clinical information on the COPD patients; AG performed the MPO and MMP assays; JEB was responsible for the overall design and conduct of the research and contributed significantly to the manuscript.
